# Cronbach’s Alpha and Semantic Overlap Between Items: A Proposed Correction and Tests of Significance

**DOI:** 10.3389/fpsyg.2022.815490

**Published:** 2022-02-10

**Authors:** Ghadah S. Alkhadim

**Affiliations:** Department of Psychology, College of Arts, Taif University, Taif, Saudi Arabia

**Keywords:** Cronbach’s alpha, classical test theory, conceptual overlap, internal consistency reliability, simulation

## Abstract

The purpose of the present study was to address the shortcomings of Cronbach’s alpha concerning the semantic overlap between items. Using an example from a motivational measure, the correction of Cronbach’s alpha was applied by partialing out the effects due to conceptual overlap. The significance of Cronbach’s alpha was tested using simulated random data derived from the measure and by estimating the confidence intervals with known and unknown distributions. The results indicated that the uncorrected conceptual overlap coefficient alpha was equal to 0.89 and 0.66 following the correction. After simulating the corrected statistical results, the distribution of alpha with random numbers had an estimate of 95%, equal to 0.41. The lower bound of the corrected alpha distribution was equal to 0.41, suggesting that the corrected alpha could easily belong to the distribution of alpha developed from simulated random numbers. Thus, the semantic overlap between items on a measure represents a significant threat to the validity of the alpha coefficient.

## Introduction

Cronbach’s alpha is a ubiquitously used index of internal consistency reliability (Cronbach, 1951; 297–334). In Google’s Scholar database, the coefficient exceeds 256,000 hits, suggesting extensive use, despite noticeable shortcomings and challenges (Shevlin et al., 2000, 229–237; Raykov, 2001, 69–76; Hayashi and Kamata, 2005, 579–586; Liu et al., 2010, 5–21) and controversies regarding computation or interpretation (Boyle, 1991, 291–294; Cortina, 1993, 98–104; Kopalle and Lehmann, 1997, 189–197; Henson, 2001, 177–189).

Based on classical test theory (Nunnally, 1978), a measured item/construct’s X score comprises two components: a true T score plus some form of error e (X = T + e). Since we rarely measure single-item constructs, unidimensional measured phenomena are often described with a single-factor model in which items contribute stochastic and white noise information. Using a three-item instrument, the one-factor model can be expressed as follows:


(Item 1) Y=1λξ1+1δ1

(Item 2) Y=2λξ2+1δ2

(Item 3) Y=3λξ3+1δ3

Each of the items y_1_–y_3_ is linked to the latent structure ξ stochastically (with λ being the correlation between the item and the latent dimension) and δ, a form of random error. Based on the above single-factor model and early work (Guttman, 1945, 255–282), Cronbach (1951) proposed the standardized alpha coefficient as a measure of internal consistency, assuming that all items contribute to the measurement of a construct, and the consistency between items is reflected through their bivariate correlation (i.e., $k^{*}\,\bar{r}$), as follows:


(1) $$\mathrm{Standardized}\,\alpha =\frac{k\,\bar{r_{i}}}{1+\left(k-1\right)\,\bar{r_{i}}}\,$$

Thus, the term $\bar{r_{i}}$ reflects the mean intercorrelation between items i_1_, i_2_…i_*k*_, and *k* is the number of items.^1^ The magnitude of the interitem correlation and the number of items are positive contributors to alpha with larger correlations, and lengthy instruments being associated with higher estimates of internal consistency reliability. However, as several researchers noted, Cronbach’s coefficient alpha is a low bound estimate to true internal consistency reliability; thus, it might seriously underestimate the true internal consistency of a measure (Osburn, 2000, 343–355).

Cronbach’s alpha requires several conditions to be met before its estimates are valid, some of which have been ignored in the literature; the pivotal ones are these: (1) interval-level data with no restriction of range (Fife et al., 2012, 862–888) without having to implement the Kuder–Richardson 20 formula; (2) linearity and homoscedasticity of errors; (3) small amounts of measurement error and correction for attenuation of both variances and covariances; (4) the same distributions between items; (5) unidimensionality; (6) absence of systematic sources of error; and (7) independence of items in terms of content. This last assumption is the focus of the present study and is described in the next section.

Moreover, several assumptions regarding Cronbach’s alpha are implicit. Among them, the most fundamental one refers to the fact that interitem correlations should reflect the relationship between independent rather than overlapping, in content, items. For example, the fear of being verbally reprimanded by parents and the fear of losing privileges from parents for failing a test are both independent facets of the construct “feared outcomes from failing a test.” In contrast, the items “fear of upsetting important others” and “fear of upsetting parents” contain significant overlap, as the fear of upsetting parents is presumably incorporated in the more global fear of “upsetting important others” (assuming that parents *are* also important others). In the latter instance, the estimation of Cronbach’s alpha will be inflated because part of the correlation between the two items would be accountable by the semantic overlap between the two items. To my knowledge, this is the first attempt to correct Cronbach’s alpha for this fundamental methodological problem.

Consequently, the present study sought to address the shortcomings of Cronbach’s alpha regarding the semantic or conceptual overlap between items. Using an example from a motivational measure, Cronbach’s alpha was estimated prior to and after correcting the mean interitem correlation due to the semantic overlap between items demonstrating the potential biases from ignoring semantic and conceptual overlap.

## Materials and Methods

### Participants and Measures

As a blueprint of real data from which simulated data would emerge, an applied data set—describing a measure of motivation—was implemented in the study. The participants included 37 university students (psychology majors) who completed the Achievement Goals Questionnaire (AGQ) (Elliot and McGregor, 2001, 501–519) for extra credit. The present study used only the performance-approach subscale (see Table 1).

**TABLE 1:** Measurement of performance approach (PAP) goals using the AGR measure (Elliot and McGregor, 2001, 501–519).

Item from performance approach goal subscale	Factor loading
1. It is important for me to do better than other students.	0.95
2. It is important for me to do well compared to others in this class.	0.93
3. My goal in this class is to get a better grade than most of the other students.	0.86

### Estimation of Semantic Overlap Between Items

The literature on semantic overlap can be traced to natural language processing, information processing, and/or artificial intelligence (Han et al., 2013). The present model implemented a metric evaluating the semantics of words rather than lexical categories using the web corpus from the Stanford WebBase project (Stanford, 2001), which included 100 million web pages from over 50,000 websites. Following exhaustive processing for removing various sources of error (e.g., non-English text, truncated text, text duplications, ineligible characters, etc.), the final corpus included three billion words of good quality English language. The similarity measure included a standard similarity index with a minimum value of zero and a maximum value of 1 (Li et al., 2003, 871–882), thus, closely resembling the measurement of a correlation coefficient.

### Correcting Cronbach’s Alpha for Semantic Overlap

Given the above description and estimation of semantic overlap, the similarity index was assumed to reflect the correlation between two items as a function of shared content, thus, violating one of the most important properties of Cronbach’s alpha, that of content independence. Thus, using the semipartial correlation formula (see Equation 2) from Pedhazur (1982), the correlation between items 1 and 2 was purified by partialing out the conceptual overlap that the second item shares with the 3d item (i.e., conceptual overlap in the form of a correlation between (Items 2 and 3) as evidenced using the UMBC semantic textual similarity service. The preference for the semipartial correlation coefficient versus the partial correlation is due to preserving the variance of the criterion variable by residualizing only the predictor variable with respect to a third variable. In other words, by preserving the total variance of the dependent variable, all semipartial correlation coefficients are comparable and on the same scale, compared to partial correlations that are expressed on a different scale, rendering interpretations difficult if not impossible. Thus, in the present context in which correlations will subsequently be used for estimating alpha, semipartial correlation coefficients are more appropriate and in the example of correlating item 1 with item 2 after partialing out the conceptual overlap of item 2 due to item 3, the equation is as follows:


(2) $$r_{1\,\left(2.3\right)}=\frac{r_{12}-s_{13}\,s_{23}}{\sqrt{1-s_{23}^{2}}}\,$$

Where r_1(2.3)_ stands for the correlation between items 1 and 2 after partialing out the effect of item 3 from item 2; r_12_ stands for the correlation between items 1 and 2; and s_12_, s_23_ reflect the conceptual overlap similarity coefficients between items 1 and 2 and 2 and 3, respectively.

Applying the actual coefficients and partialing out the conceptual overlap between the second and third variables for each of the three pairs of correlation coefficients as they would be computed in alpha (i.e., correlation between items 1,2, items 1,3, and items 2,3) we obtain the following triplet r_1(2.3)_, r_1(3.2)_, r_2(3.1)_; however, because the correlation between r_(1.2)_ is equal to r_(2.1)_, by extending the idea of the semipartial correlation coefficients which are not symmetric we have more combinations from which alpha can be computed such as r_1(2.3)_, r_2(1.3)_, r_1(3.2)_, r_3(1.2)_, r_2(3.1)_, r_3(2.1)_. Thus, because e.g., r_1(2.3)_ is not equivalent to r_2(1.3)_ in order to increase the sensitivity by which alpha will be computed I propose averaging the asymmetric semipartial correlation coefficients to form the three triplets as follows: (a) r_1(2.3)_, r_2(1.3)_, (b) r_1(3.2)_, r_3(1.2)_, and (c) r_2(3.1)_, r_3(2.1)_, which result in the following estimates:


(3a) $$r_{1\,\left(2.3\right)}=\frac{r_{12}-s_{13}\,s_{23}}{\sqrt{1-s_{23}^{2}}}=\frac{0.869-\left(0.602\right)\,\left(0.327\right)}{\sqrt{1-0.327^{2}}}=0.710$$

(3b) $$r_{2\,\left(1.3\right)}=\frac{r_{21}-s_{23}\,s_{13}}{\sqrt{1-s_{13}^{2}}}=\frac{0.869-\left(0.327\right)\,\left(0.602\right)}{\sqrt{1-0.602^{2}}}=0.841$$

(4a) $$r_{1\,\left(3.2\right)}=\frac{r_{13}-s_{12}\,s_{23}}{\sqrt{1-s_{23}^{2}}}=\frac{0.712-\left(0.724\right)\,\left(0.327\right)}{\sqrt{1-0.327^{2}}}=0.503$$

(4b) $$r_{3\,\left(1.2\right)}=\frac{r_{31}-s_{32}\,s_{12}}{\sqrt{1-s_{12}^{2}}}=\frac{0.712-\left(0.327\right)\,\left(0.724\right)}{\sqrt{1-0.724^{2}}}=0.689$$

(5a) $$r_{2\,\left(3.1\right)}=\frac{r_{23}-s_{12}\,s_{13}}{\sqrt{1-s_{13}^{2}}}=\frac{0.667-\left(0.724\right)\,\left(0.602\right)}{\sqrt{1-0.602^{2}}}=0.289$$

(5b) $$r_{3\,\left(2.1\right)}=\frac{r_{32}-s_{31}\,s_{21}}{\sqrt{1-s_{21}^{2}}}=\frac{0.667-\left(0.602\right)\,\left(0.724\right)}{\sqrt{1-0.724^{2}}}=0.335$$

In the above estimates, the partialed-out coefficients reflected the semantic overlap correlations produced by the University of Maryland (https://ebiquity.umbc.edu/blogger/2013/01/10/word-and-phrase-similarity/), Baltimore County’s (UMBC) system (Han et al., 2013). Table 2 shows the correlation matrices with and without partialing out the conceptual overlap between items.

**TABLE 2:** Correlation matrix before and after partialing out the conceptual overlap between items.

Item from performance approach goal subscale	Item 1	Item 2	Item 3
1. It is important for me to do better than other students.	*1*	*0.710*	*0.503*
2. It is important for me to do well compared to others in this class.	0.868	1	*0.289*
3. My goal in this class is to get a better grade than most of the other students.	0.712	0.667	1

*Estimates below the diagonal are original bivariate correlations; estimates above the diagonal (in italics) are bivariate correlations after partialing out conceptual overlap using semipartial correlations.*

## Results

### Uncorrected and Corrected Estimates of Cronbach’s Alpha

Equations 6, 7 show the uncorrected and corrected estimates of Cronbach’s standardized coefficient alpha following correction for overlap. The latter uses the means pairs 3a and 3b, 4a and 4b and 5a and 5b as shown above to estimate the mean correlation of a triplet.


(6) StandardizedCronbach′sα=Originalk⁢r¯1+(k-1)⁢r¯=3⁢(0.749)1+(3-1)⁢(0.749)=2.2472.498=0.899

(7) StandardizedCronbach′sα=correctedk⁢r¯1+(k-1)⁢r¯=3⁢(0.561)1+(3-1)⁢(0.561)=1.6832.122=0.793

As expected, the corrected alpha estimate of internal consistency, accounting for conceptual overlap is 0.793 compared to 0.899 of the original estimate, thus, moving from an excellent to a good estimate. Trying to move away from the heuristic of cutoff points (e.g., acceptable alpha > 0.80), we simulated the distribution of alpha coefficients that were derived from the empirical data using reshuffling (see Leontitsis and Pagge, 2007, 336–340) to estimate the distribution of alpha with random data. The simulation is described below.

### Contrasting Uncorrected and Corrected for Conceptual Overlap Alpha Coefficients

To test the hypothesis of equivalence of Cronbach alpha estimates using uncorrected and corrected for semantic overlap correlations I employed the Wald Chi-square test using Mplus 8.7. The estimate of the alpha coefficient using the uncorrected data was equal to 0.899 with a standard error of 0.035. Using the model test command in Mplus, I constrained the observed alpha estimate of 0.899 to be equivalent to 0.793, the corrected one for conceptual overlap. If this constrain is supported by the data, then a non-significant Wald chi-square test would be obtained suggesting that the observed value of 0.899 is no different from 0.793, and the opposite. Results indicated support of the alternative hypothesis that the alpha estimate of 0.899 was significantly different from that of 0.793 [χ^2^(1) = 10.274, *p* = 0.001] pointing to the salient differences between the two Cronbach alpha estimates. Thus, correcting alpha for conceptual overlap was associated with a significantly lower alpha estimate compared to ignoring content overlap.

### Simulating Alpha Coefficient With Random and Empirical Data

To test the hypothesis that the corrected alpha internal consistency estimate (of 0.793) was no different compared to what would be observed from random data, I conducted a Monte Carlo simulation. Following the work of Leontitsis and Pagge (2007), I estimated the distribution of the alpha coefficients derived from random data using the initial estimates provided by the sample. The simulation involved reshuffling and intermixing the values of all three columns (X_1_, X_2_, and X_3_) to estimate the sampling distribution of the alpha coefficient with random numbers. Alpha estimates were computed for 10,000 datasets generated using a mean of zero and a variance of 0.35 as observed from the empirical data. Results indicated that the mean of the alpha population distribution of random numbers was equal to −0.0006 and the 95% Confidence Intervals (C.I.s) ranged between −0.368 and 0.366. Thus, an alpha coefficient up to 0.366 represents a plausible estimate of internal consistency reliability with random data.

**FIGURE 1:**
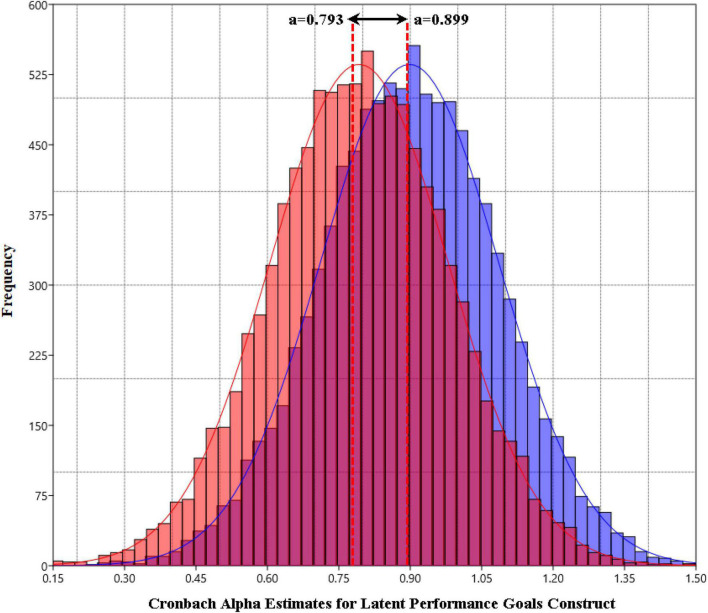
Population distributions of coefficient alpha with means of 0.750 (corrected for conceptual overlap alpha) and 0.899 (uncorrected alpha estimates). Population distributions were simulated using the Monte Carlo facility in Mplus 8.7.

Using the 0.793 point-estimate of the corrected for conceptual overlap alpha estimate and a variance equal to 0.035 (the observed variance with the observed data) I simulated the population distribution of alpha coefficients using 10,000 replicated samples (Figure 1). The mean from that population distribution was equal to 0.793 and that point estimate was surrounded by 95% C.I.s ranging between 0.409 and 1.00. Given the upper bound 2.5% cutoff value of the random data an alpha coefficient of 0.366 could still be a valid member of the population distribution. Using the corrected alpha population distribution, the 2.5% low bound estimate of alpha was 0.409. Thus, the corrected for conceptual overlap alpha coefficient could be as low as 0.409 but not as low as one that could be estimated with merely random numbers. Similarly, when contrasting the random data alpha population distribution to that of the uncorrected alpha, the low bound estimate (i.e., C.I.95%) of the latter was 0.531. Given that the upper 2.5% estimate of the population alpha distribution with random numbers was 0.366, one can rule out the hypothesis that an alpha coefficient of 0.899 could be an outcome from estimating a coefficient with random numbers.

## Discussion

The present study sought to address the shortcomings of Cronbach’s alpha regarding semantic overlap between items of an assumed unidimensional measure. A measure of goal orientations (Elliot and McGregor, 2001, 501–519) revealed that the items shared a significant amount of underlying content. Using a correction on Cronbach’s alpha based on semantic overlap by using similarity index statistics, the re-estimated alpha was lower by 0.106 units. The new alpha estimate was compared to both, a simulated distribution of Cronbach’s alpha values that originated from random numbers and the 95% C.I.s of the known F distribution as well as the Wald test. The results indicated that the corrected alpha estimate was significantly different from the original estimate thus, correcting for conceptual overlap was associated with a significantly lower estimate of alpha. However, the corrected alpha estimate was also significantly different from alpha estimates that would be a function of random numbers. Thus, ignoring the semantic overlap between items of a measure significantly inflated the alpha and invalidated its meaning and interpretation by violating the basic assumption of conceptual between-item overlap put forth by Cronbach.

The present study had several limitations: First, my attempt to partial out conceptual overlap using a similarity index might have underestimated or overestimated the actual effects under certain circumstances. For example, the words used colloquially rather than in literature or other texts might be underestimated using a web corpus (UMBC, 2013). Second, other ways of defining similarity must be investigated further by including judges and panels of experts on linguistics to evaluate conceptual overlap. Nevertheless, the present study demonstrated one method for dealing with the salient problem in the estimation of Cronbach’s alpha, which seriously invalidates its estimation, interpretation, and use. If the conceptual overlap is substantial in measures evaluating psychological states and traits, the concept of internal consistency reliability must be revisited. The present study advances our understanding of a potential problem of a yet-unknown magnitude and provides a solution and direction for appropriately using and evaluating alpha. Certainly, more research is required to compare and contrast various methodologies for assessing conceptual overlap. Another direction of research involves evaluating the magnitude of conceptual overlap in educational/psychological assessments.

## Data Availability Statement

The raw data supporting the conclusions of this article will be made available by the authors, without undue reservation.

## Ethics Statement

The studies involving human participants were reviewed and approved by National and Kapodistrian University of Athens. The patients/participants provided their written informed consent to participate in this study.

## Author Contributions

GA developed the literature, designed the methodology, did the analysis, and reported the results and discussed them.

## Conflict of Interest

The author declares that the research was conducted in the absence of any commercial or financial relationships that could be construed as a potential conflict of interest.

## Publisher’s Note

All claims expressed in this article are solely those of the authors and do not necessarily represent those of their affiliated organizations, or those of the publisher, the editors and the reviewers. Any product that may be evaluated in this article, or claim that may be made by its manufacturer, is not guaranteed or endorsed by the publisher.

## Footnotes

1 Later, Cronbach (1951) corrected the positive bias that the number of items exerts on the coefficient by adopting the Spearman–Brown formula (Brown, 1996) and proposed alternative formulations (Cronbach and Gleser, 1964).
